# A Case Report of Severe Bromadiolone Poisoning: Blood Toxicology Levels and Correlation to Clinical Toxicity

**DOI:** 10.7759/cureus.99159

**Published:** 2025-12-13

**Authors:** Man Ching Patrick Kong, Elizabeth Ming Jing Tan, R Ponampalam, Yi Ju Yao

**Affiliations:** 1 Emergency Medicine, Sengkang General Hospital, Singapore, SGP; 2 Emergency Department, Singapore General Hospital, Singapore, SGP; 3 Accident and Emergency/Clinical Toxicology Department, Singapore General Hospital, Singapore, SGP; 4 Toxicology, Health Science Authority, Singapore, SGP

**Keywords:** bromadiolone, emergency department, luteal cyst rupture, poisoning, superwarfarin poisoning, toxicology

## Abstract

Bromadiolone is a long-acting anticoagulant rodenticide (“superwarfarin”) that can cause severe, prolonged coagulopathy. Its occurrence in Singapore is rare, with limited reported cases. We report a toxic cluster involving three household members with confirmed bromadiolone poisoning. The index patient, a 49-year-old woman, presented with abdominal pain, hematuria, and hemoperitoneum secondary to a ruptured corpus luteal cyst. Laboratory tests revealed profound coagulopathy (prothrombin time (PT) >180 s, international normalized ratio (INR) unmeasurable) with warfarin-like factor deficiencies. Serum bromadiolone level was 150 ng/mL; the other two household members had levels of 250 ng/mL and 450 ng/mL, respectively. The patient underwent surgical intervention, received intravenous vitamin K every six hours, blood product transfusions, and supportive therapy. She recovered fully after 10 days and was discharged on tapering oral vitamin K. This cluster highlights the diagnostic challenge of superwarfarin poisoning in the absence of a clear exposure history. Literature suggests toxicity can occur at serum bromadiolone levels as low as 117 ng/mL. Blood testing is preferred over urine due to bromadiolone’s high lipophilicity and long half-life. Early recognition, targeted toxicology testing, and prompt initiation of vitamin K therapy are essential. In regions with low incidence, superwarfarin poisoning should be suspected in patients with unexplained bleeding and markedly prolonged coagulation profiles. Cluster detection warrants urgent public health investigation to identify and control potential sources.

## Introduction

Bromadiolone is regarded as one of the superwarfarins that can lead to life-threatening coagulopathy. Superwarfarins are long-acting, vitamin K antagonist anticoagulant rodenticides that are very potent to induce coagulopathy and have very long half-lives [1]. Superwarfarin poisoning is relatively uncommon in Singapore locally, and we present a case of bromadiolone poisoning that presented to our emergency department (ED) with coagulopathy and intra-abdominal bleeding. Two other teenage household members were presented to another Children’s ED with bleeding diatheses and subsequently confirmed to have bromadiolone poisoning, forming a toxic cluster [2]. We present the serum bromadiolone levels for this cluster and review the literature on its clinical correlation. This cluster could raise physicians’ awareness to consider toxicology cause in patients with similar presentations. The bromadiolone levels in this cluster could help predict the clinical course and prognosis and guide management in future cases.

The case has been presented as an abstract poster in the 22nd Scientific Congress of The Asia Pacific Association of Toxicology (APAMT 2024) .

## Case presentation

**Figure 1:**
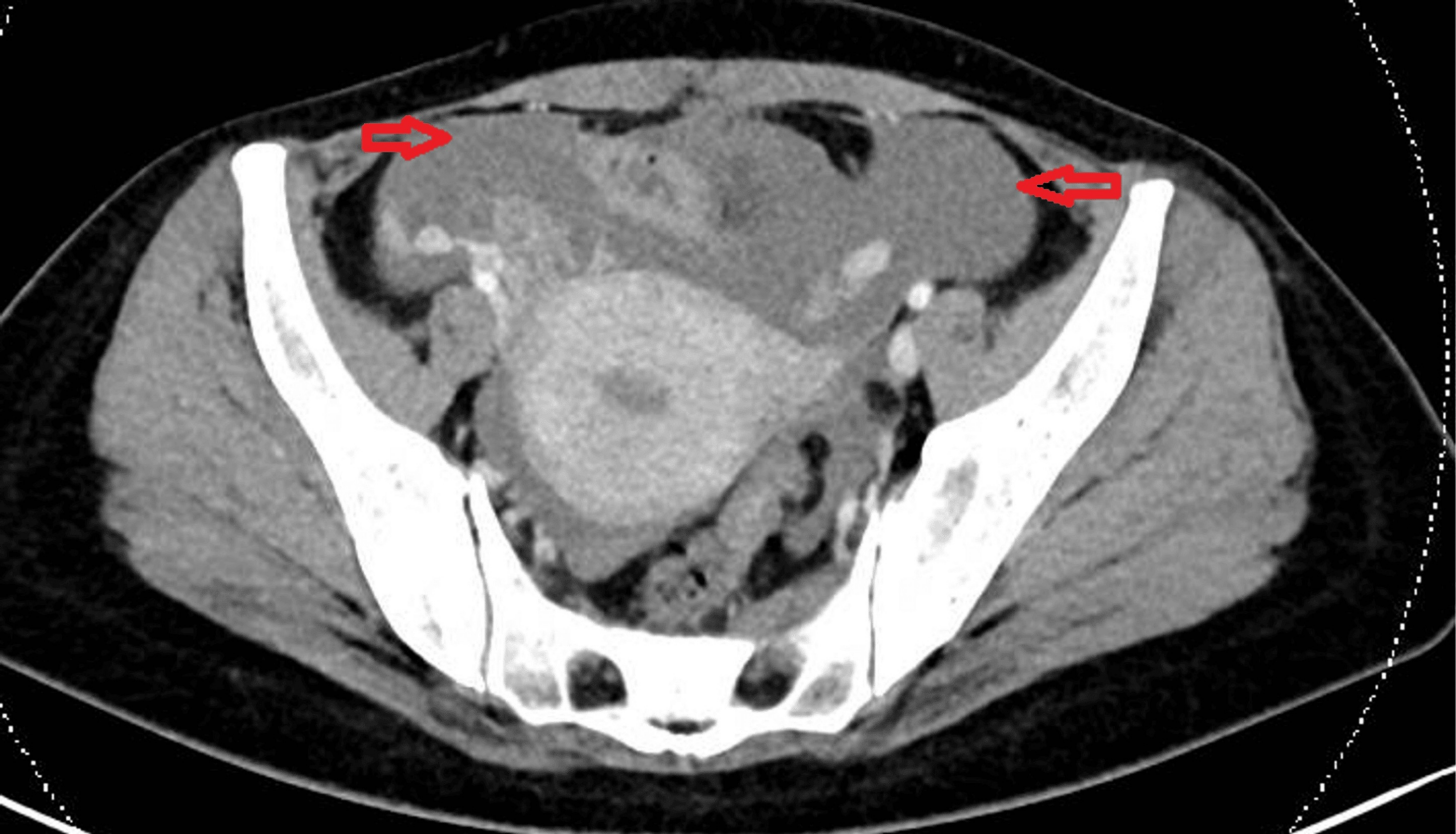
CT abdomen and pelvis of the patient showing large amount of hemoperitoneum (red arrows). The CT scan was performed at around two hours after the initial presentation.

A 49-year-old Chinese woman presented to the EDs with a four-day history of constant abdominal pain, associated with non-bloody vomiting and diarrhea, and persistent gross hematuria. The patient had no past medical history and no past records suggestive of any bleeding diathesis. On examination, she was afebrile, with a heart rate of 103 beats per minute, borderline blood pressure of 88/57 mmHg, a respiratory rate of 23 breaths per minute, and saturation of 100% on room air. She had pallor, with abdominal tenderness over her right iliac fossa region, without guarding or rebound. Point-of-care ultrasound of the abdomen showed free fluid. Her blood investigations showed anemia with a normal platelet count, but with profound coagulopathy (Table 1). Urinalysis showed RBC >2250/uL, WBC 2160/uL, epithelial cells 180/uL, and nitrite negative. Subsequent factor assays revealed warfarin-like deficiency with markedly reduced levels of factor II, VII, IX, and X. Computed tomography of the abdomen and pelvis (CTAP) was done in the ED and showed large-volume hemoperitoneum, with the likely source being a ruptured right corpus luteal cyst (Figure 1).

**Table 1:** Initial blood investigations of the patient

Test	Result	Reference range
Hemoglobin (g/dL)	6.6	12.0-16.0
Hematocrit (%)	20.4	36.0-46.0
White blood cell count (x 10^9^/L)	8.22	4.00-10.00
Albumin, serum (g/L)	39	35-50
Total bilirubin, serum (µmol/L)	7	3-21
Alkaline phosphatase, serum (U/L)	63	35-104
Alanine transaminase, serum (U/L)	18	<=35
Aspartate transaminase, serum (U/L)	18	<=35
Platelet count (x 10^9^/L)	224	140-400
Prothrombin time (s)	>180	9.6-11.9
Activated partial thromboplastin time (s)	88	24.4-35.2
Prothrombin assay (%)	22	80-160
Factor V assay (%)	133	70-170
Factor VII assay (%)	13	40-180
Factor VIII assay (%)	398	5-200
Factor IX assay (%)	10	40-200
Factor X assay (%)	21	60-160
Factor XI assay (%)	116	70-200
Factor XII assay (%)	73	30-200

Due to the largely abnormal coagulation profile and the bleeding source from a gynecological origin, the patient was transferred to a tertiary hospital with hematology and gynecology support. Prior to transfer, the patient received one unit of red blood cells and one unit of fresh frozen plasma.

After the hospital transfer, further history revealed that two other household members also presented to another local hospital for abnormal bleeding [2]. Case 2 was a 19-year-old female with worsening abdominal pain for four days and one day of gross hematuria, with hemodynamic instability (heart rate of 135 beats/minute, blood pressure of 81/56 mmHg), with a deranged coagulation profile of hemoglobin 4.7 g/dL, platelet count 200 x 10^9^/L, activated partial thromboplastin time (APTT) 109.4 seconds, and prothrombin time (PT) >120 seconds. Computed tomography showed a ruptured left ovarian cyst with extensive hemoperitoneum. Case 3 was a 9-year-old boy who presented with a four-day history of frequent epistaxis, with stable hemodynamics. His blood investigations showed hemoglobin 9.8 g/dL, platelets 275 x 10^9^/L, APTT 75.2 seconds, and PT >120 seconds. As the cases were all related and suffered similar coagulopathic issues, a common source of poisoning was suspected. Blood toxicology confirmed all three patients had bromadiolone poisoning (Table 2). Cases were reported to the local Health Sciences Authority (HSA).

**Table 2:** Serum bramadiolone levels of all three cases

Case	Bromadiolone level (ng/mL)
Case 1	150
Case 2	250
case 3	450

Our patient underwent diagnostic laparoscopy, right cystectomy, and peritoneal washout around 20 hours after admission. Prior to the surgery, the patient received fresh frozen plasma 15 ml/kg, cryoprecipitate 10 units, and intravenous (IV) vitamin K 10 mg before coagulopathy was deemed sufficiently corrected for the procedure. During the course of her hospital stay, she received ongoing supportive treatment with blood product transfusions, IV vitamin K, and B12/folate and iron replacement. Postoperatively, the patient was first continued with IV vitamin K 15 mg every six hours. As international normalized ratio (INR) monitoring was persistently less than 1.4 for five days, vitamin K was administered orally 20 mg two times a day on postoperative day 6 and tapered down to oral vitamin K 10 mg two times a day for three weeks upon discharge. The patient was admitted to the hospital for a total of 10 days and was discharged well. After discharge, she was on an ongoing tapering dose of oral vitamin K, with 10 mg two times a day for three weeks, then 10 mg per day for two weeks, and then 5 mg per day for two weeks. Cases 2 (19-year-old female) and 3 (9-year-old boy) were also discharged well with oral vitamin K after hospital stays of 13 days and eight days, respectively (detailed management for Cases 2 and 3 was described by Tan et al. [2]).

Follow-up laboratory tests of case 1 two weeks after discharge showed a normal coagulation profile with a platelet count of 199 x 10^9^/L, INR 1.01, and PT 10.6 seconds. The latest laboratory test results before the patient was lost to follow-up, which were around two months after initial presentation, showed a platelet count of 242 x 10^9^/L, PT 10.5 seconds, and INR 1.0.

## Discussion

This case series illustrates the challenges in diagnosis and management of superwarfarin poisoning without a clear exposure history [1,3,4,5], which could potentially lead to delayed diagnosis and suboptimal treatment. Superwarfarin poisoning is uncommon in Singapore, with the only reported case locally being the two patients in this cluster [2].

A systematic approach to investigate coagulopathic patients with considerations to exclude potential toxins should be utilized. This was readily achieved, and the blood specimens were sent to the toxicology laboratory with confirmation of bromadiolone. This allows for treatment planning, anticipating the course of the poisoning with an appropriate regimen using vitamin K and blood products, and regular monitoring of INR. Surgical interventions were planned strategically to reduce the risks of ongoing bleeding and postoperative bleeding complications.

Specific toxicology screening request for superwarfarins revealed bromadiolone. Further quantitation by liquid chromatography with Orbitrap mass spectrometry gave bromadiolone levels of 150 ng/ml; the other two related cases had bromadiolone levels of 250 ng/ml and 450 ng/ml, respectively. Similar or higher levels (239-750 ng/ml) were reported in the blood/serum samples of a number of bromadiolone-exposed patients who all survived after treatment [6-9]. Literature review has shown that consumption of 0.17 mg/kg of bromadiolone could cause toxicity [10]. In addition, serum bromadiolone levels as low as 117 ng/mL could cause toxicity [11]. There is no documented lethal dose for humans, while the LD50 for rodents and non-rodents was found to be 1-3 mg/kg [12]. Serum bromadiolone level less than 10 ng/mL was found to have a normal coagulation profile [10]. However, there were cases reported to be symptomatic and have deranged coagulation profiles (INR 1.55 to unclotted) with much lower serum bromadiolone level (3.9 to 74.5 ng/mL) in the condition of accidental recurrent consumption [13]. Rat baits with 0.005% bromadiolone are available in Singapore for rodent control, and the possibility of accidental or intentional poisoning cannot be ruled out. In the event of suspected bromadiolone poisoning, blood is the preferred specimen over urine for toxicology screening due to its high lipophilicity and long elimination half-life (three to six days in the early phase and 10-24 days in the later phase). Animal studies showed that bromadiolone is excreted in urine only in very small amounts [14].

The most important clinical features of superwarfarin poisoning are hemorrhages and a bleeding tendency [1]. Symptoms and signs include bleeding from the mucosa, gastrointestinal tract, genitourinary tract, hemoptysis, menorrhagia, and non-bleeding symptoms such as headache, abdominal pain, joint pain, and flank pain [1,3,15,16]. Interestingly, co-existing thrombosis and bleeding have been reported [17,18]. Central nervous system effects have also been reported [7,19]. PT and INR are the investigations of choice to identify the coagulopathy caused by superwarfarin [20]; to confirm superwarfarin poisoning, quantitative analysis of superwarfarins is the test of choice [1,10]. In the case we presented, albeit rare in the local context, there was suspicion of superwarfarin poisoning due to the similar presentation of bleeding diatheses for all patients who lived in the same household. With the largely deranged PT and INR, the patients were supported with red blood cells and fresh frozen plasma transfusions before the tertiary hospital transfer. For rapid correction of severe coagulopathy from vitamin K antagonism, both FFP and four-factor prothrombin complex concentrate (4-factor PCC) have been suggested [1,15]. There were no studies with direct comparison of both blood products’ effectiveness, but in a randomized trial, Goldstein et al. found that, combined with vitamin K1, rapid INR reduction was achieved in 55% of patients in the four-factor PCC group, compared to 10% of patients in the FFP plus vitamin K1 group [21].

The treatment of superwarfarin poisoning includes vitamin K1 therapy and rapid correction of coagulopathy [1,15,22]. GI decontamination and multi-dose activated charcoal were found to be unsuccessful [14,23,24]. The optimal frequency of vitamin K1 administration was suggested to be every six to hours [25]. Our patient received IV vitamin K every 6 hours, with adjuncts of blood product, folate/B12, and iron replacement. Surgical intervention was also employed very early (within 24 hours) for bleeding control.

On discharge, our patients were put on a tapering dose of vitamin K therapy with regular hematology clinic follow-up with PT/INR monitoring. Currently, there are no established guidelines for the stopping of vitamin K therapy after known superwarfarin poisoning. It is noted that termination of vitamin K therapy based on normal PT/INR while the patient is still on vitamin K can be dangerous, with a study showing that patients who received vitamin K therapy could have normalized PT/INR but raised plasma long-acting anticoagulant rocenticide level upon hospital discharge [26-27]. While no established guidelines, many centers employ an approach of stopping vitamin K therapy followed by measurement of coagulation parameters at 48-72 hours for normalization [3].

## Conclusions

Superwarfarin poisoning can be difficult to diagnose, especially in countries with low incidence. A high index of suspicion of poisoning should be considered in patients with abnormal bleeding and severely deranged coagulation profiles. Prompt confirmation with relevant toxicology testing aids risk assessment and strategic planning of therapeutic interventions. Situational awareness and early recognition of such poisoning clusters should trigger an immediate public health response, searching for potential sources of contamination by activation of the relevant authorities to investigate and mitigate a possible public health hazard.
